# Notes from Dental Practice

**Published:** 1869-01

**Authors:** 


### ARTICLE II.

### *Notes from Dental Practice.*

*Extraction of Difficult Teeth.* 1st. *Superior Cuspidati.* Nature of Case.—The right cuspidatus perfectly sound, crown short and thick, root corresponding to crown with firm alveolar attachment.

*Operation.*—All attempts with the ordinary form of incisor forceps were of no avail, owing to the concave edges of the beaks rotating on the neck of the tooth, and preventing the rotary motion from being applied in connection with the outward and inward motions. The gum lancet was then freely used, not alone for separating the gum and periosteum about the neck of the tooth, but that the alveolar attachment might, at least, be partly severed by carrying the edge of the blade as far up between the alveolar process and root of the tooth as was possible. A pair of duck-bill incisor forceps was then applied, and the edges of the beaks pressed high up on the root inclosing a small portion of the process which was cut through to the tooth.

By placing the ball of the thumb in the bifurcation between the handles close to the joint, the pressure of the beaks of the instrument was so regulated as to prevent it from acting as an excising forceps.

The object in using this duck-bill form was not only to obtain a higher hold upon the root, but that the rotary motion might be used in connection with the outward and inward motions, as the edges of the beaks of the instrument were pressed so far into the root as to prevent them from rotating; at the same time care was taken to so govern this pressure, by the means above referred to, as to prevent the root from being cut through.

The outward and inward motions were used as little as possible, in order that the labial and palatine walls of the cavity might be preserved, for, owing to the strong alveolar attachments of these teeth, it frequently happens that considerable portions of these walls are fractured and brought away; whereas if the rotary force is the one most depended upon, we may succeed in breaking up the attachments of the root without injury to the alveolar process.

In the extraction of the superior incisor teeth, the rotary motion only should be used for the reason just given, but owing to the root of a cuspidatus being more or less flattened. it is, in the majority of cases, necessary to apply the outward and inward motions in connection with the rotary motion. Although the fracture of the walls of the alveolar cavities may result in no serious injury, yet where these walls are preserved, the absorption, which follows as a necessary consequence of the removal of teeth, occurs much more regularly, leaving the ridge in a more favorable form for the insertion of artificial substitutes.

Where one of the adjoining teeth is to be removed at the same time as the cuspidatus, the better plan is not to attempt the extraction of this latter until after that of the lateral incisor or first bicuspid, as an open cavity with a thin septum next to the cuspidatus will render its removal a much easier operation.

*The left superior cuspidatus of the same mouth.* Nature of Case.—The crown badly decayed and very frail, the caries extending along the palatine surface for a considerable distance beyond the margin of the gum.

*Operation.*—The crown of the the tooth being so nearly separated from the root, it was deemed best, as a first step in the operation, to remove the remaining portion of the crown, which was readily done by means of a pair of excising forceps. A vertical incision was then made in the gum immediately over a portion of the root of the tooth, and the flaps raised from the alveolar process. The portion of the process over the root being thus exposed, a pair of duck-bill incisor forceps was applied by carrying the cutting edges of the beaks as high up as the incision in the gum extended, and the bone cut through to the root of the tooth.

By such means a secure hold was obtained, but when the force was applied, although every care was taken to avoid cutting through the root, yet owing to its extreme brittleness this accident occurred and a portion only came away. It was necessary then to carry the forceps still higher up, in order to obtain a hold upon the remaining portion of the root, and a way was made for this by passing the edge of the gum lancet between the gum and alveolar process at the upper part of the vertical incision, without extending this incision beyond its original limit.

The long, narrow, curved duck-bill root forceps was substituted for the incisor forceps before used, and by cutting through the process the remaining portion of the root was seized and removed.

2nd. *Inferior 1st Molar of the Right Side.* Nature of Case—A crowded arch, and at least one fourth of the crown of the molar destroyed by caries attacking its anterior approximal surface. The posterior approximal surface of the second bicuspid also affected, but capable of being filled; its crown, however, projected over into a portion of the space formed by the action of caries on that of the molar.

*Operation.*—The first and only difficulty which presented itself upon a careful examination, was the removal of the molar without injury to the adjoining bicuspid, as the space between the second molar and this tooth was not sufficient for the remaining portion of the neck and the roots of the first molar to pass. But as the preparation of the cavity in the second bicuspid would necessitate the cutting away of the thin and irregular edges in order to properly fill it, some space was thus gained.

After freely lancing the gum in order to carry the edges of the beaks of the extracting instrument down to, and, if possible, a short distance under the margin of the alveolar process, the form of forceps adapted to the removal of inferior molars from the right side of the jaw was applied, with the expectation of being able to remove the tooth from between the adjoining ones in the usual manner, or, in case the space was not sufficient for this, to open a passage for it by pressing out the buccal wall of the cavity after the periosteal attachment was broken by the inward and outward motions.

These motions proved effectual in breaking this connection, but all attempts failed to raise the tooth directly from its cavity or to press it out at the side although considerable movement could be given to it. Suspecting, therefore, that the difficulty was owing to the divergence of the roots, or to their apexes approaching each other after first diverging on leaving the neck so as to inclose a considerable thickness of process between them, it was considered best to use the splitting forceps for the purpose of separating the roots. The sharp ends of the blades of this instrument were then inserted so low down on the neck of the tooth as to cause them to enter the bifurcation between the roots, and pressure applied to the handles. By such means the roots were separated, and then removed one at a time with a pair of inferior bicuspid forceps, by applying the force according to the inclination of each root.

The difficulty was found to be owing to both roots being so curved at their ends in the form of hooks, as to inclose a considerable thickness of process which was firm enough to resist all attempts to raise them from their cavities in the ordinary manner, or from the side towards the cheek while yet joined together.

*Insertion of an Artificial Crown on the Roots of a Superior Bicuspid Tooth.* Nature of Case.—The crown of the bicuspid was so badly decayed as to offer no hope of its preservation by filling; the roots in a healthy condition and the patient averse to wearing a plate.

*Operation.*—The remaining portion of the crown was first removed by means of the excising forceps and pivot file, care being taken in the use of the forceps, that small portions only of the tooth substance should be detached at a time, in order to save fracturing the roots, and also to prevent destructive inflammation from being incited. The operation was commenced with the hope of finding one root only, but after the removal of a portion of the crown, a nerve instrument revealed the presence of two. Such being the case, and the neck of the tooth fully covered by the gum, no more of this portion was removed by the file than would allow the edge of the base of the artificial crown to be hidden by the free margin of the gum, as the removal of the entire neck would have separated the roots.

The exposed surface of the neck was filed level with the margin of the gum, instead of concave as is usual in cases of teeth with a single root, and each pulp canal enlarged sufficiently to admit a wooden pivot of ordinary size, both as regarded diameter and length. The edge of this surface towards the cheek was slightly beveled in order that the edge of the base of the artificial crown could be carried up under the margin of the gum, while at the same time the neck of the tooth was left thick enough over the bifurcation of the roots to hold these latter firmly together. A plaster model obtained from a wax impression of the exposed surface of the neck and the adjoining teeth, enabled us to prepare the artificial crown in the following manner, without the presence of the patient being necessary during the entire operation: A plain plate cuspid tooth was ground to fit accurately the exposed surface of the neck, and a gold backing or lining attached by a method which answers well in all cases where it is necessary that the backing at the base should be of considerable thickness. The tooth was first backed or lined with a piece of thin platina plate, so extended beyond the base of the tooth as to allow of its being bent back at a right angle with the portion covering the palatine surface; the height of this bent portion corresponded to the thickness of the backing when finished. The tooth was then invested in a mixture of asbestos and plaster, with the backing, however, free and fully exposed. Scraps of gold plate were then melted, by means of the blow pipe, on this platina backing, the gold rising at the base as high as the bent portion of the platina, and at the apex on a level with the pins of the tooth which penetrated the platina backing without being bent over or cut off. After removing the tooth from the investment, the base of the crown and backing was coated with a thin layer of wax and adjusted to the exposed surface of the neck over the pulp canals. The impressions in the wax indicated the position of the two pivots which were to be attached to the base of the backing on the crown, and the next step was to prepare these pivots, one for each of the roots. These pivots were formed from gold wire filed square and tapering towards the ends, and the plaster model materially assisted in placing them in their proper position on the base of the backing attached to the tooth, sealing wax being used to retain them until the tooth was a second time invested.

After soldering the pivots to the backing, the crown was ready to be attached to the roots, which was done as follows: Wooden pivots were inserted into the pulp canals of both roots and cut off level with the surface of the neck of the tooth; then holes were drilled in the centre of each of these pivots to a depth corresponding to the length of the square metallic pivots. These square pivots having sharp corners, barbs were formed on each with a sharp knife, the points of the barbs having a direction towards the crown. The operation was then completed by forcing the square and pointed metallic pivots into the holes made in the wooden pivots of the pulp canals in the roots, as far as was necessary to bring the base of the crown in contact with the exposed surface of the neck.

Instead of wooden pivots in the pulp canals, the attachment of the artificial crown to the roots can be made by means of small square tubes of the same taper as the pivots, fitted into the enlarged pulp canals, and held firmly in place by being packed about with gold foil. The pivots designed to enter these tubes, should be made of gold alloyed with platina in order that they may have the elasticity required; and each should consist of two halves soldered together at the end where they are attached to the base of the backing of the crown, but open for some distance from their free ends. When both crown and roots are prepared the split end of each pivot is slightly opened, and on pressing them into the square tubes in the pulp canals they act as springs and thus keep the crown firmly fixed.

